# Impact of Integrating Social and Health Home Care Services in Catalonia: A Retrospective Cohort-Based Two-Year Study

**DOI:** 10.5334/ijic.8909

**Published:** 2025-05-16

**Authors:** Emili Vela, Joan Carles Contel, Anna Vila, Sebastià J. Santaeugènia, Rosa Suñol, Jordi Amblàs-Novellas, Conxita Barbeta, Aina Plaza, Pilar Hilarión

**Affiliations:** 1 Àrea de Sistemes d’Informació, Servei Catalàde la Salut, Barcelona, Spain; 2 Digitalization for the Sustainability of the Healthcare System (DS3), IDIBELL, Barcelona, Spain; 3 Integrated Social and Health Care Program, Department of Health and Department of Social Rights, Generalitat de Catalunya, Barcelona, Spain; 4 General Directorate of Health Planning, Department of Health, Generalitat de Catalunya, Barcelona, Spain; 5 Central Catalonia Chronicity Research Group (C3RG), Centre for Health and Social Care Research (CESS), University of Vic-Central University of Catalonia (UVIC-UCC), Barcelona, Spain; 6 General Directorate of Personal Autonomy and Disability, Department of Social Rights, Generalitat de Catalunya, Barcelona, Spain; 7 Intermediate Care Director, Parc Sanitari Sant Joan de Déu, Sant Boi de Llobregat, Spain; 8 Avedis Donabedian Research Institute (FAD), Spain; 9 Universitat Autònoma de Barcelona, Barcelona, Spain; 10 Health Services Research Network on Chronic Diseases (REDISSEC), Spain; 11 Network for Research on Chronicity, Primary Care, and Health Promotion (RICAPPS), Spain

**Keywords:** health and social care services utilization, home care services, impact of integration, integrated care, social care, cost-effectiveness

## Abstract

**Introduction:**

This study aimed to evaluate the impact of integrating social and health home care services (HCSs) on institutionalization, survival, and utilization of health and social care services and associated expenditures.

**Methods:**

Retrospective study including all individuals who initiated social HCSs for dependent people in Catalonia during 2018–2019 with a paired case-control, pre/post design (differences-in-differences), using integrated data from the Autonomy and Dependency Care System database and the Catalan Health Surveillance System. Individuals were categorized based on their perceived level of integration in the residing areas (high level: Case Group, and low level: Control Group).

**Results:**

We included 4381 cases and 13143 matched controls. HCS integration decreased the risk of institutionalization in a nursing home by 19.5% and the length of stays in any center by 7.7%. Integration increased day centers’ use by 23.7% and primary care service utilization by 3.0%, and also reduced associated social expenditures after HCS initiation by 19.1% and overall social and health expenditures by 2.6%.

**Conclusion:**

Integration of social and health HCSs resulted in a shift from institutionalization services to primary care services and day care centers, prolonging recipients’ lives in their homes.

## Introduction

Home care is defined as the care provided at individuals’ homes, including informal care provided by non-professional caregivers and formal care provided by professionals from the public and private sectors [1]. Home care is regarded as a sustainable strategy to meet individuals’ needs while enabling their independent lives in their homes and has become a priority in long-term policies [2]. Demographic changes in developed countries with increasingly older populations and decreased availability of informal care have resulted in a high demand for these services, which are expected to increase during the following decades, posing organizational and financial challenges to service providers [3].

To ensure an efficient, individual-centered, effective delivery of home care, different professional teams encompassing multiple disciplines need to be coordinated [2,4]. However, home care services (HCSs) are often provided independently by social and healthcare systems, with different organizations and structures of services, resulting in a fragmented delivery (Social Care Institute for Excellence 2019). Moreover, in many countries, the private sector provides social HCSs, outsourced by the public sector due to its limited capability of responding to the high demand. Effective home care coordination requires an integrated delivery, which can be achieved at different levels, to prevent overlaps and gaps in service provision and improve quality and efficiency [1]. International agencies and governments have acknowledged this need and have developed plans to promote integrated care [5,6,7].

In Catalonia, the Department of Health provides health HCSs, including activities for disease prevention and health promotion involving primary health professionals, such as physicians, nurses, and social workers; hospital-at-home care, based on hospital healthcare professionals actively treating patients that otherwise would need acute care in a hospital; and end-of-life care through multidisciplinary and specialized teams [8]. Social services are co-financed and organized by the local government and are provided by not-for-profit and for-profit private providers. They cover different areas, in which dependent individuals are considered a priority [9]. Dependency was defined as a person’s need for help to perform basic activities of daily living (ADL) due to age, illness, and/or disability, and linked to lack or loss of physical, mental, intellectual, or sensory autonomy [10,11]. Individuals with the official recognition of dependency through a formal proof assessment have granted access to a specific package of economic allowances and services according to users’ choices from a comprehensive portfolio. These services include social HCSs, mostly operated by private providers, telecare, day centers, and residential care, in addition to service-linked allowances, such as that for caregivers. Users may change the service package according to their degree of dependency to meet their specific needs [12].

The Department of Health and Department of Social Rights developed joint plans to promote the transformation of the existing care models into an integrated care delivery model [13,14,15] and, in 2020, they developed an integrated person-centered home care program: the Catalan model for integrated health and social home care [16,17]. This model aims to deliver patient-centered, multidimensional, comprehensive services through a tailored, shared care plan generated by a multidisciplinary team of professionals [17].

The initiatives promoting home-based care integration of the Government of Catalonia have not been evaluated at the population level and may provide valuable information for implementing the Catalan model for integrated health and social home care between 2020 and 2026 [17]. This retrospective study included individuals from Catalonia who initiated social HCSs for people with the official recognition of dependency between 2018–2019. The study aimed to evaluate the impact of the integration of HCSs on institutionalization, survival, and utilization of health and social care services and associated expenditures. We used a natural experimental approach with a paired case-control pre/post design (differences-in-differences design). In the context of current regulations that protect sharing information between sites, obtaining integrated health and social care data is challenging. Nonetheless, this study collected information provided by both health and social care services integrated in a joint database from the Autonomy and Dependency Care System database, from the Department of Social Rights, and the Catalan Health Surveillance System, from the Department of Health.

## Methods

### Study Design and Population

This was a retrospective study including all individuals who initiated social HCSs for dependent people in Catalonia during 2018–2019, representing a population-based natural experiment, with a paired case-control, pre/post design (differences-in-differences). The term “social HCSs” is used throughout the manuscript as equivalent to social HCSs for dependent people (i.e., with the official recognition of dependency). We used a differences-in-differences design [18] to evaluate the impact of the integration of social and health HCSs on institutionalization, survival, and utilization of health and social care services and associated expenditures. In Catalonia, primary and community care is delivered through primary health centers (PHCs), whereas social home care private providers deliver Social Care Services (SCSs), co-financed by the Department of Social Rights, local governments, and, in many cases, co-payment by users, based on their incomes. The individuals who initiated HCSs were categorized as cases and controls based on the degree of health and social HCS integration of the SCSs and PHCs of their area of residence: individuals residing in areas with a high level of integration were considered to receive integrated care and were included in the Case Group, and those residing in areas with a low level of integration were considered to receive non-integrated care and were included in the Control Group with a Case:Control ratio of 1:3. Individuals from areas with an intermediate level of integration were not analyzed.

A diagram of the study design and selection of populations is included in Figure S1. The differences-in-differences design allowed us to minimize bias and maximize the ability to infer causality by evaluating the differences between cases and controls before and after the intervention (i.e., social HCS initiation), following the recommendations of the “Institut Català d’Avaluació i Polítiques Públiques” (Ivàlua) (Catalan Institute of Evaluation and Public Politics) [19]. This study design requires a robust definition and pairing of controls, as described below. This study was conducted in accordance with the Ethical Principles for Medical Research Involving Human Subjects of the Helsinki Declaration and the local Personal Data Protection Law (LOPD 15/1999), and was approved by the Research Ethics Committee of the University of Vic-Central University of Catalonia (UVIC-UCC) (reference number 176/2021).

### Social and Health HCSs

The package of social HCSs for dependent people includes help with household needs, such as cleaning, food preparation, and laundry, as well as personal care, such as hygiene, dressing, and undressing. These services are offered at variable levels according to the person’s needs and degree of dependency and are provided by not-for-profit and for-profit private providers.

Health HCSs are provided by primary care teams from PHCs, including primary care physicians, nurses, and health social workers.

### Assessment of the Integration Level

The different areas in Catalonia providing social and health HCSs (i.e., PHCs and SCSs) were classified according to the level of HCS integration (low, intermediate, and high). Integration was defined based on the implementation of the five core components of an integrated care model. These components had been previously established in the Catalan model for integrated health and social home care [16,17] and included in the evaluation framework [20], summarizing the main quality indicators of integrated social and health HCS offered in Catalonia and reported in the literature. Their selection entailed a consensus process with the participation of the community, families, users, professionals, and policy makers. Moreover, in line with the previous official documents, the Catalan Government established that integration of HCS should be evaluated according to the five components: 1) Individualized integrated social and health assessment, 2) Unique individual care plan, 3) Shared protocols between health and social services, 4) Coordination between multidisciplinary social and healthcare teams, and 5) Integrated service portfolio with joint social and healthcare projects in the home environment (Table S1).

The integration level was determined based on a previous study conducted between December 2020 and June 2021 assessing the overall perception of social workers within SCSs and PHCs regarding the implementation of the five core components using a screening questionnaire [20,21]. Details on the questionnaire and classification of areas are summarized in the supplementary methods (Supplementary file).

### Selection and Pairing of Cases and Controls

For pairing of cases and controls (1:3 ratio), we used the Nearest Neighbor Method, which selects the most similar controls (i.e., the best controls) for each case. Sex and dependency degree were required to be the same in cases and controls; other variables considered for pairing were age, multimorbidity index according to the GMA stratification, healthcare expenditures during the previous year, income level, and type of municipality.

### Data Sources

This study used two administrative databases that register social and healthcare data, allowing the integration of social, morbidity, and health information. Sociodemographic and social services utilization data were obtained from the “Sistema per l’autonomia i l’atenció a la dependència” (SIDEP) (Autonomy and Dependency Care System) central registry. Morbidity, mortality, and utilization of healthcare services data were obtained from the Catalan Health Surveillance System (CHSS) from the Department of Health. The databases are described in detail in the supplementary methods.

### Variables

This study considered sociodemographic, clinical, healthcare, and social service utilization, as well as dependency-associated variables. The sociodemographic variables considered were age, sex, income level, and type of municipality.

Clinical variables were diagnoses, coded following the International Classification of Diseases, ninth revision, Clinical Modification (ICD-9-CM) [22], and the morbidity-associated risk, stratified using the GMA. The GMA is a clinically validated variable registered in the electronic and shared clinical records of Catalonia that assigns a numerical morbidity index summarizing all comorbidities [23,24,25]. Additional clinical variables were the identification of patients as complex chronic patients (CCPs) and advanced chronic patients (ACPs) as previously reported [26].

Dependency was categorized into three degrees: Level I (moderate), Level II (severe), and Level III (very severe) according to the regulations of the Social Rights Department of the Government of Catalonia [11]. The social HCS used (personal care and household support) and intensity (hours of care per month) were also considered; data regarding informal care was not available and was not considered. The two types of social HCS, public service or service-linked economic allowance (“prestació econòmica vinculada” [PEV]), were considered. Data regarding other social services, including non-professional caregiver allowance, telecare, and day center, and healthcare services utilization were collected from the corresponding databases. Associated expenditures were calculated applying the standard costs of each service provided by Catalonia’s health and social authorities for each year [27].

### Statistical Analysis

Basic statistical methods are summarized in the supplementary methods. A formal sample size calculation was not applicable in this study, as it considered all individuals starting social HCSs within the study period.

Changes in services utilization and expenditures before and after social HCS initiation were estimated using a mixed-effects generalized linear model that considered correlations between repeated measures obtained from one individual, which was considered a random effect. The models used were logistic regression for dichotomic variables, Poisson regression for services utilization, and lognormal regression for expenditures, adjusted by age, sex, income level, morbidity, dependency level, healthcare expenditure during the year before social HCS initiation, and type of service (public service and PEV). The reference values were those of controls before social HCS initiation and were set at 1 and 0 for services utilization and expenditures, respectively. These models allowed to assess the statistical significance of changes and provided a relative risk with a feasible application in terms of percentage of variation. Furthermore, differences between groups before and after social HCS initiation were used to calculate a double difference. A two-year survival analysis was performed using the Kaplan-Meier method and the Cox Proportional Hazards model to compare events occurring throughout time, including mortality and institutionalization in a nursing home. The statistical significance threshold was set at a bilateral α = 0.05. All analyses were performed using the R statistical package (version 4.0.3).

## Results

### Characteristics of Cases and Controls

**Figure 1:**
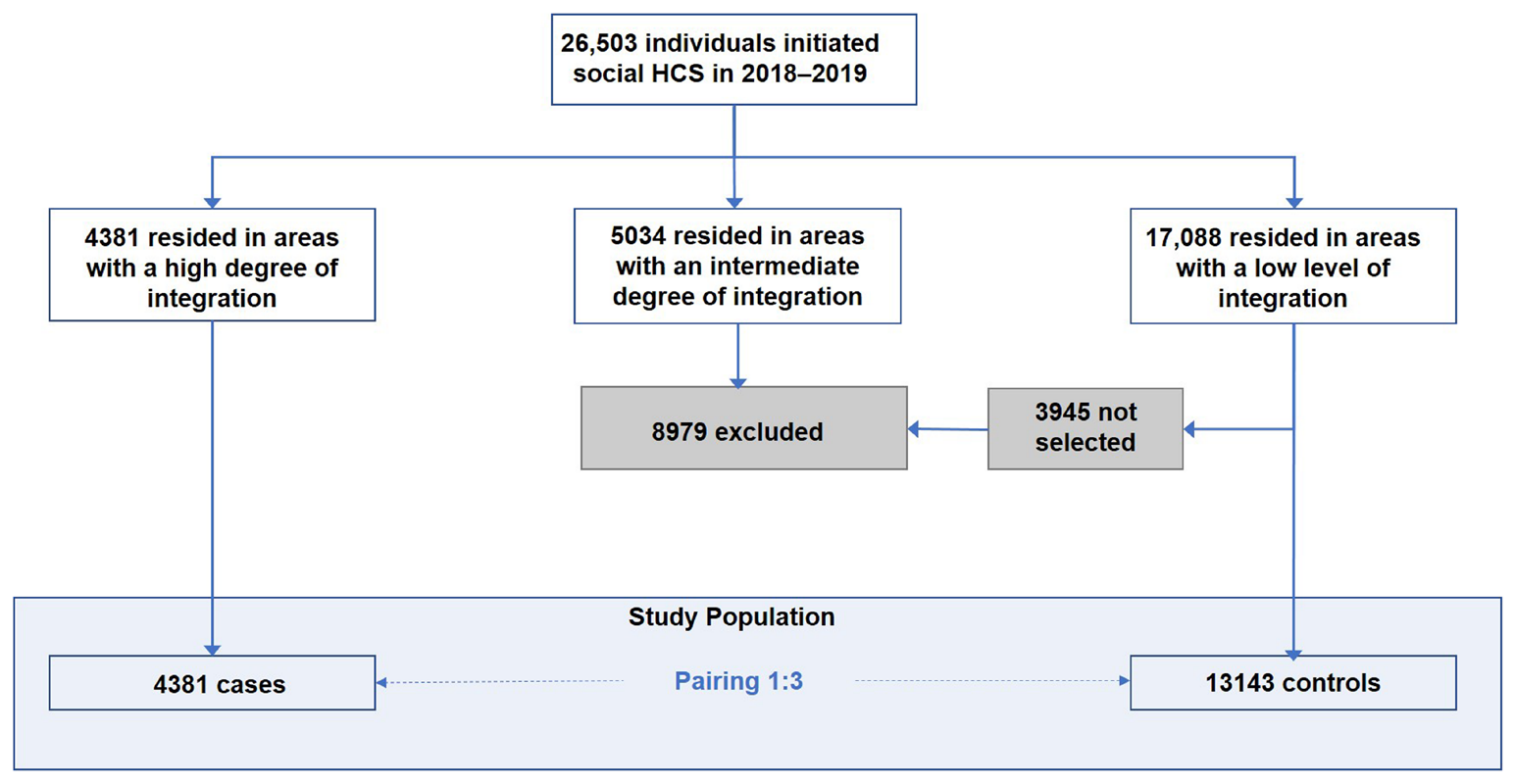
Flow diagram of the study population and allocation into Case and Control Groups.

Of the 26 503 individuals who initiated social HCSs in Catalonia during 2018–2019, 4381 lived in areas with a perceived high level of integration according to the results of the screening questionnaire, and were included in the Case Group, and 13143 matching individuals residing in areas with a low level of integration were included in the Control Group, from a total of 17 088 eligible control individuals (Figure 1).

Overall, the individuals included were old persons with a moderate-high morbidity-associated risk. As expected, the variables considered for the pairing of cases (i.e., sex and dependency level) were similar between groups, despite the statistically significant results of the paired analysis due to the large sample size (Table 1). All remaining variables were similar, except for social HCSs, which were more frequent in the form of services and not economic allowances in cases compared with controls. Differences between cases and controls in the other variables considered for pairing were marginal and not significant.

**Table 1:** Comparison of characteristics of cases and controls.


	CASES n = 4381	CONTROLS n = 13143	*P-VALUE* ^a^

Sex, n (%)			1

Men	1468 (33.5)	4404 (33.5)	

Women	2913 (66.5)	8739 (66.5)	

Dependency degree, n (%)			1

I	2734 (62.0)	8202 (62.4)	

II	1226 (28.0)	3678 (28.0)	

III	421 (9.61)	1263 (9.61)	

Age (years)			<0.001

Mean (SD)	79.4 (14.2)	79.7 (13.8)	

Median (IQR)	83 (76–87)	83 (77–87)	

Income level, n (%)			0.511

High	2 (0.05)	0 (0.00)	

Medium	497 (11.3)	1.485 (11.3)	

Low	3622 (82.7)	10 891 (82.9)	

Very low	260 (5.93)	767 (5.84)	

GMA index			<0.001

Mean (SD)	27.1 (14.3)	27.0 (13.9)	

Median (IQR)	24.4 (16.7–35.3)	24.4 (16.7–35.0)	

GMA stratification, n (%)			0.276

Baseline risk	24 (0.55)	58 (0.44)	

Low risk	227 (5.18)	633 (4.82)	

Moderate risk	1631 (37.2)	4998 (38.0)	

High risk	2499 (57.0)	7454 (56.7)	

Delivery of social home care, n (%)			<0.001

Service	3564 (81.4)	10 158 (77.3)	

Economic allowance	817 (18.6)	2985 (22.7)	

Kind of municipality, n (%)			<0.001

Urban	3702 (84.5)	11 128 (84.7)	

Semi-rural	394 (8.99)	1167 (8.88)	

Rural	285 (6.51)	848 (6.45)	

Healthcare expenditure in the previous year (Euros/person/year)			<0.001

Mean (SD)	5545 (7871)	5369 (7414)	

Median (IQR)	3112 (1482–6593)	3062 (1509–6292)	


IQR, interquartile range; GMA, Adjusted Morbidity Groups; SD, standard deviation. ^a^McNemar and Wilcoxon paired-data.

### Survival in Cases and Controls

**Figure 2:**
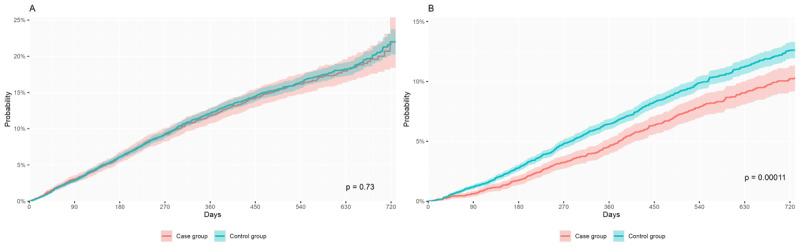
Probability of **(A)** survival and **(B)** institutionalization up to 2 years after initiation of social HCSs of cases and controls (2018–2019 period).

The probability of death of social and health HCS recipients after social HCS initiation was similar regardless of the level of HCS integration, reaching 22% at two years in cases and controls (Figure 2A). Accordingly, HCS recipients with integrated services (i.e., cases) had the same risk of dying compared to those without integrated services (i.e., controls) (HR 0.990 [95% CI 0.897–1.093]) after adjusting for confounding variables.

### Impact of Integration on Institutionalization and Other Social Services Utilization Before and After HCS Initiation

The integration of HCSs resulted in a significantly decreased probability of institutionalization in a nursing home after adjusting for confounding variables. Two years after social HCS initiation, the probability of institutionalization was 10.2% in cases and 12.3% in controls (Figure 2B). Accordingly, the HR of institutionalization at two years was 0.805 (95% CI 0.716–0.904) in cases vs. controls, indicating that users of integrated services had a 19.5% decreased risk of institutionalization at two years after social HCS initiation.

Regarding the use of the different services included in social HCSs for dependent people portfolio (Table 2), the intensity of HCSs for personal care was similar regardless of integration, with a mean (SD) of 17.7 (11.4) hours per month in cases and 17.9 (11.8) in controls. However, household services showed an overall higher use in cases than controls: a higher proportion of cases (36.6%) used HCSs for household needs compared to controls (32.1%), and for 0.3 more hours (Table 2). Regarding changes in other social services during the year before and after social HCS initiation, the use of non-professional caregiver allowance decreased a 2.8% more in cases than controls, whereas the use of day centers increased a 0.6% more in cases than controls (Table 2).

**Table 2:** Changes in the utilization of social services by cases and controls during the year before and after home care services initiation (2018–2019 period).


	CONTROLS			CASES			DOUBLE DIFFERENCE	RATIO^a^	95% CI
	
	BEFORE	AFTER	CHANGE	BEFORE	AFTER	CHANGE			

**Social HCSs**									

Personal care (hours/person/month), *mean*	0	17.9	17.9	0	17.7	17.7	–0.2	1.012	0.959–1.066

Household (hours/person/month), *mean*	0	2.4	2.4	0	2.7	2.7	0.3	1.050	1.018–1.081

Household, %	0	32.1	32.1	0	36.6	36.6	4.5	1.382	1.337–1.428

**Other social services, %**									

Non-professional caregiver allowance	18.4	13.3	–5.1	22.8	14.9	–7.9	–2.8	0.734	0.658–0.819

Telecare	2.9	38.6	35.7	3.1	38.7	35.6	–0.1	0.933	0.822–1.058

Day center	2.2	3.6	1.4	2.2	4.2	2	0.6	1.237	1.016–1.506


CI, Confidence interval; HCSs, home care services. ^a^Adjusted by sex, age, dependency level, income level, morbidity, and previous kind of service and healthcare expenditure.

**Figure 3:**
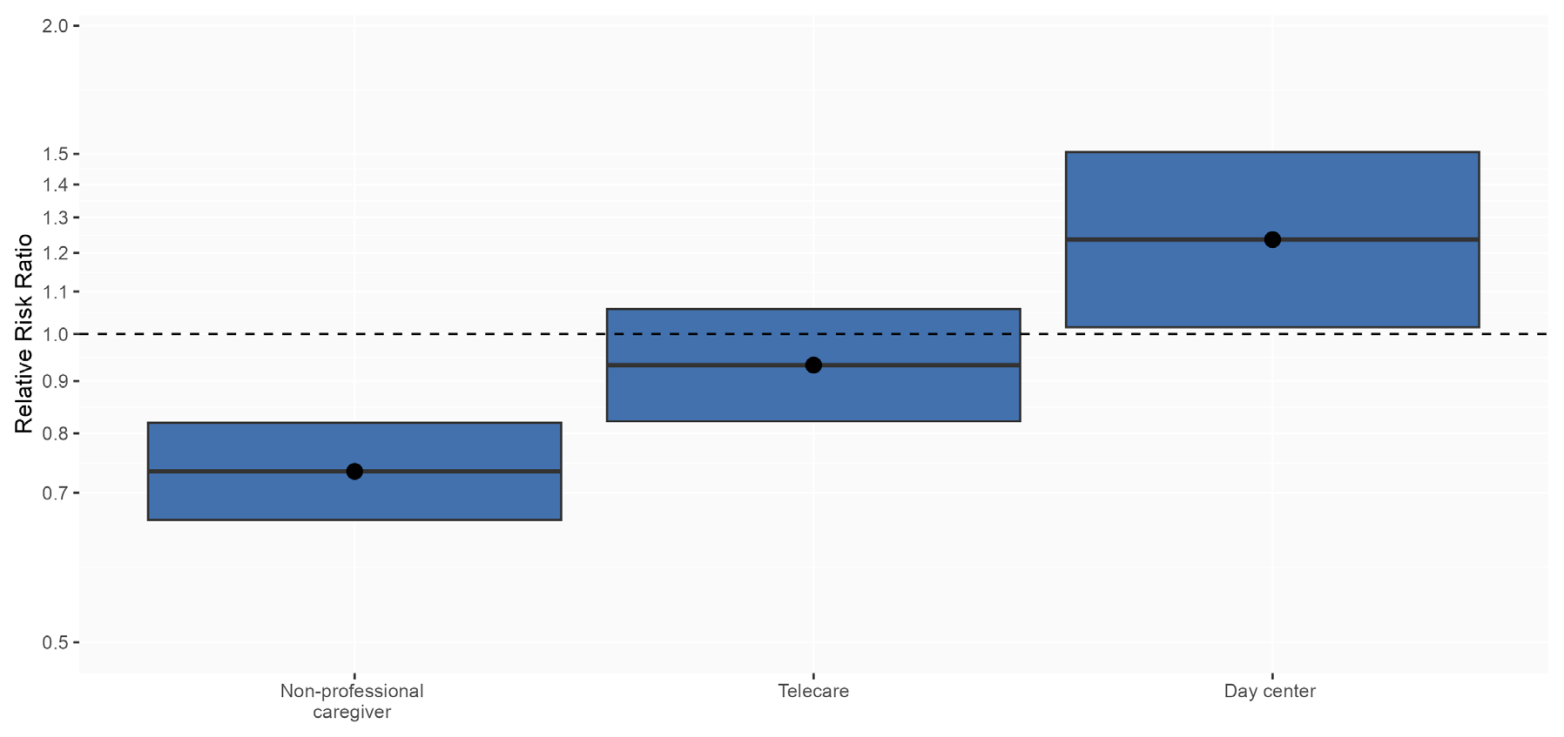
Impact of the integration of social and health home care services on the utilization of social services. A ratio of relative risks adjusted by age, sex, dependency level, income level, morbidity and previous kind of services and healthcare expenditures is presented (2018–2019 period). The reference rate (set at 1) was that of controls before social HCS initiation. The lines across each box represent the ratio of changes in utilization rates before and after social HCS initiation between cases and controls, and the blue boxes represent the 95% confidence interval. The dashed line at 1.0 indicates no change and the blue boxes above and below the line indicate significantly increased and decreased utilization between groups, respectively.

The evaluation of changes in the utilization of social services adjusted by age, sex, dependency level, income level, morbidity and previous kind of services and healthcare expenditures showed consistent results. Recipients of integrated care (cases) were 38.2% more likely to use HCSs household than controls. Moreover, they were 23.7% more likely to use day centers and 26.6% less likely to use the allowance for non-professional caregivers than users with non-integrated services (controls) (Figure 3 and Table 2).

### Impact of Integration on Healthcare Services Utilization Before and After Social HCS Initiation

Changes in healthcare services utilization before and after social HCS initiation are summarized in Table 3. Visits to physicians and nurses increased to a higher extent in cases than controls. In contrast, visits to social workers decreased similarly in both groups, likely due to initiation of social HCS, which decreased the need for consultation with social workers in PHCs regardless of the integration level. At-home and remote primary care visits increased more in cases than controls, and those held in primary care centers decreased less in cases than controls. Moreover, the integration of services impacted hospitalizations, emergency hospitalizations, and emergency care visits, with a slightly higher increase in cases vs. controls. The total duration of hospitalizations and admissions in any health or social care center was used to measure the effectiveness of HCSs. Integration substantially impacted their duration, reducing the increase observed after social HCS initiation by 9.3 days/month per 100 individuals. Non-emergency transportation and duration of admissions in intermediate care centers decreased more in cases than controls. Regarding medication, integration curbed the increase after social HCS initiation (Table 3).

**Table 3:** Changes in the utilization of healthcare services by cases and controls before and after social HCS initiation (2018–2019 period). Monthly rates per 100 individuals.


	CONTROLS			CASES			DOUBLE DIFFERENCE	RATIO^a^	95% CI
	
	BEFORE	AFTER	CHANGE	BEFORE	AFTER	CHANGE			

Overall visits to primary care services	211.3	217.7	6.4	206.0	219.2	13.2	6.8	1.030	1.012–1.050

Primary healthcare professional									

Physician visits	105.8	105.3	–0.6	100.5	103.5	3.0	3.6	1.034	1.007–1.061

Nurse visits	91.7	101.5	9.8	91.1	104.6	13.4	3.6	1.034	1.006–1.063

Social worker visits	13.8	10.9	–2.8	14.4	11.1	–3.3	–0.4	0.971	0.899–1.049

Location of visits									

Primary care center	126.9	122.4	–4.5	124.1	122.4	–1.7	2.9	1.018	0.994–1.043

Home	41.7	51.9	10.2	39.7	52.3	12.6	2.3	1.062	1.020–1.105

Remote	42.7	43.4	0.7	42.2	44.5	2.3	1.6	1.035	0.993–1.079

Hospitalizations	5.4	5.7	0.3	6.0	6.7	0.7	0.4	1.048	0.936–1.172

Emergency hospitalizations	3.8	4.3	0.5	4.2	5.4	1.2	0.7	1.122	0.986–1.277

Visits to emergency services	15.4	15.9	0.5	16.2	17.7	1.5	1.0	1.047	0.977–1.121

Outpatient consultations	38.3	32.2	–6.0	40.7	35.9	–4.8	1.2	1.040	0.994–1.087

Medications	830.9	837.4	6.5	838.1	843.5	5.5	–1.0	0.994	0.984–1.003

Non-emergency transportation	42.7	39.5	–3.3	42.7	38.0	–4.7	–1.4	0.945	0.906–0.985

Intermediate care center admissions (days)	79.0	55.9	–23.1	81.6	56.6	–25.0	–1.9	0.977	0.945–1.011

Total duration of hospitalization/institutionalization^b^ (days)	119.3	144.8	25.5	121.5	137.7	16.1	–9.3	0.923	0.905–0.943


CI, confidence Interval; HCSs, home care services. ^a^Adjusted by sex, age, dependency level, income level, morbidity, and previous kind of service and healthcare expenditure. ^b^Hospitalizations and institutionalizations in any health or social care center, including acute care, mental health, intermediate care, and institutionalization centers, are considered.

**Figure 4:**
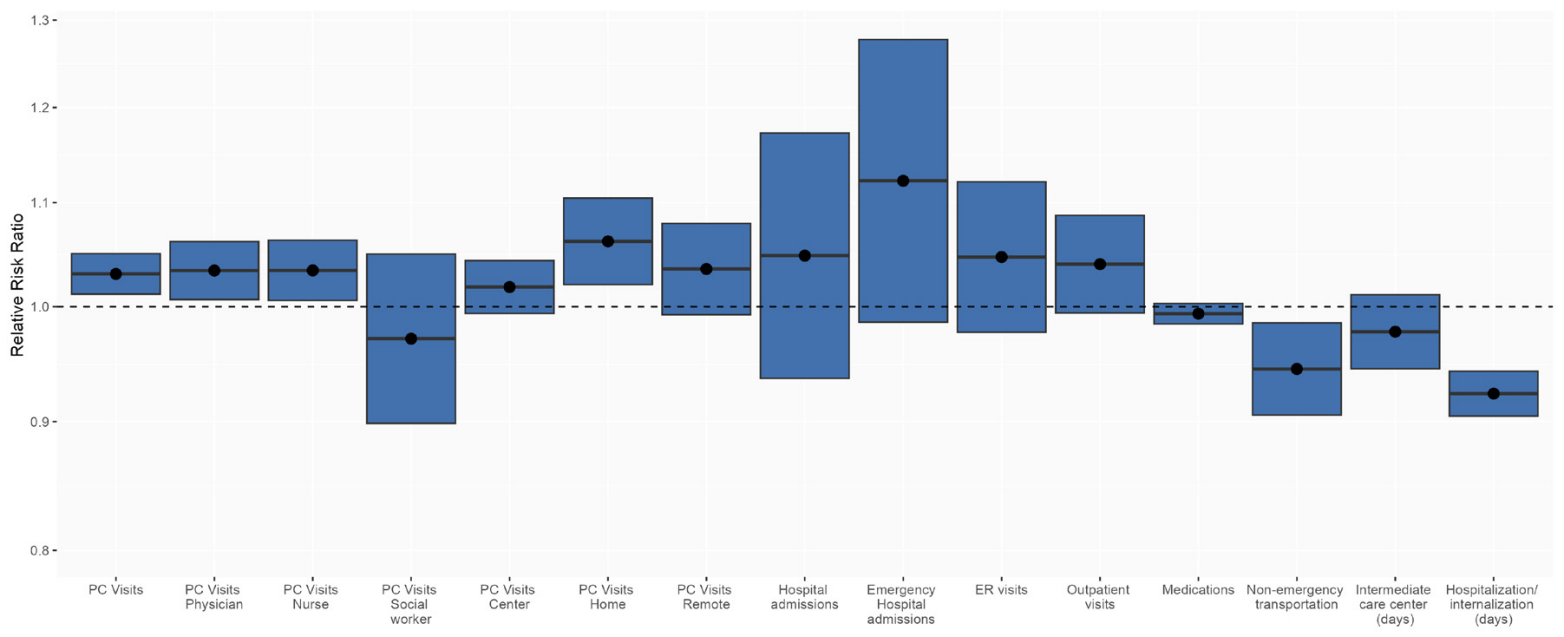
Impact of the integration of social and health home care services on the utilization of healthcare services. A ratio of relative risks adjusted by age, sex, dependency level, income level, morbidity, and previous kind of services and healthcare expenditures is presented (2018–2019 period). The reference rate (set at 1) was that of controls before social HCS initiation. The lines across each box represent the ratio of changes in utilization rates before and after social HCS initiation between cases and controls, and the blue boxes represent the 95% confidence interval. The dashed line at 1.0 indicates no change, and the blue boxes above and below the line indicate significantly increased and decreased utilization between groups, respectively.

The evaluation of changes adjusted by age, sex, dependency level, income level, morbidity, and previous kind of services and healthcare expenditures confirmed the previous results. Of all primary care services analyzed, visits to physicians and nurses increased by 3.4% and home visits by 6.2% in cases vs. controls, with a 3.0% overall increase in primary care services. Non-emergency transportation decreased by 5.5%, and the total duration of any hospitalization/institutionalization decreased by 7.7% (Figure 4 and Table 3).

### Changes in Social and Health Care Services-Associated Expenditures

**Figure 5:**
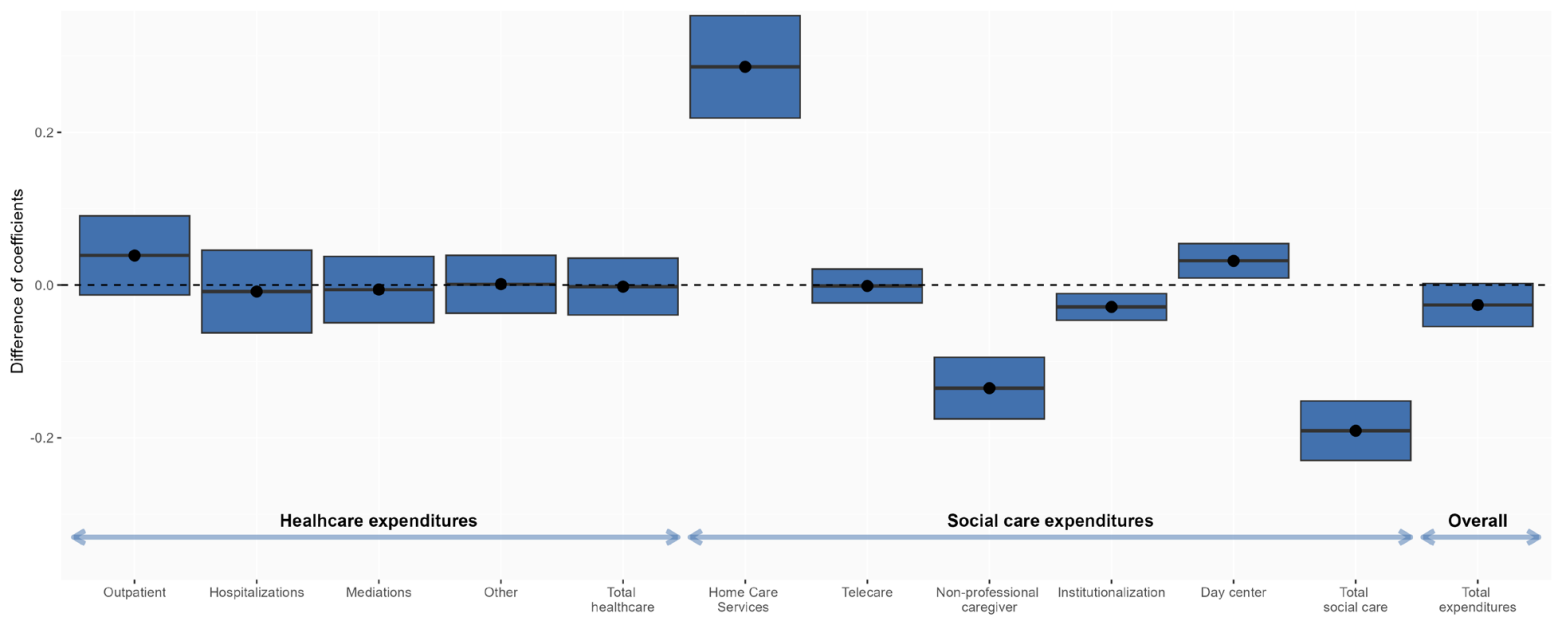
Impact of the integration of social and health home care services on changes in social and healthcare-associated expenditures. The difference of coefficients, adjusted by age, sex, dependency level, income level, morbidity, and previous kind of services and healthcare expenditures is presented (2018–2019 period). The reference expenditure (set at 0) was that of controls before social HCS initiation. The lines across the boxes represent the differences in coefficients of expenses before and after social HCS initiation between cases and controls, and the blue boxes represent the 95% confidence interval. The dashed line at 1.0 indicates no change, and the blue boxes above and below the line indicate significantly increased and decreased expenses between groups, respectively.

Changes in healthcare-associated expenditures before and after the initiation of social HCSs were similar regardless of the integration of services, with only marginal differences (Table 4). Overall, total monthly expenditures per person were €9.8 lower in cases than controls. A multiple regression analysis adjusted by age, sex, dependency level, income level, morbidity, and previous kind of services and healthcare expenditures confirmed the lack of changes in healthcare-associated expenditures (Figure 5). However, total social expenditures decreased by 19.1% in cases compared to controls, resulting in a 2.6% decrease in total expenditures, which was close to statistical significance (Figure 5). Social services with significantly decreased associated expenditures were non-professional caregiver allowance (–13.5%) and institutionalization (–2.9%) (Figure 5 and Table 4).

**Table 4:** Changes in expenditures associated with social and healthcare services utilization in cases and controls before and after social HCS initiation (Euros/person/month).


	CONTROLS			CASES			DOUBLE DIFFERENCE	COEFICIENTDIFFERENCE^a^	95% CI
	
	BEFORE	AFTER	CHANGE	BEFORE	AFTER	CHANGE			

**Healthcare-associated expenditures**									

Outpatient	112.4	112.4	0.0	111.1	113.3	2.2	2.2	0.039	–0.013–0.090

Hospitalizations	200.3	186.0	–14.3	229.4	209.5	–19.9	–5.6	–0.008	–0.063–0.046

Mediations	124.5	126.2	1.6	127.0	131.3	4.3	2.6	–0.006	–0.049–0.037

Other	46.9	48.4	1.5	41.2	40.8	–0.3	–1.9	0.001	–0.037–0.039

Total healthcare-associated expenditures	484.1	477.1	–7.1	508.6	496.9	–11.7	–4.7	–0.002	–0.039–0.035

**Social care-associated expenditures**									

Home care services Dependency	0.0	326.4	326.4	0.0	329.5	329.5	3.2	0.286	0.219–0.353

Telecare	0.4	5.0	4.6	0.4	5.0	4.6	0.0	–0.001	–0.023–0.021

Non-professional caregiver	47.0	38.3	–8.6	56.4	42.7	–13.8	–5.1	–0.135	–0.175 – –0.095

Institutionalization	4.9	28.9	24.0	2.3	20.8	18.5	–5.5	–0.029	–0.046 – –0.011

Day center	6.6	10.4	3.8	6.5	11.7	5.2	1.4	0.032	0.009–0.054

Total social care-associated expenditures	59.0	394.0	334.9	66.0	395.8	329.8	–5.2	–0.191	–0.230 – –0.152

**Total expenditures (healthcare and social)**	543.1	871.0	327.9	574.7	892.7	318.1	–9.8	–0.026	–0.054–0.002


CI, confidence interval. ^a^Adjusted by sex, age, dependency level, income level, morbidity, and previous kind of service and healthcare expenditure.

## Discussion

In this retrospective study including individuals who initiated social HCSs for dependent people in Catalonia during 2018–2019, we found that integration of social and health HCSs decreased the probability of institutionalization in a nursing home by 19.5%, prolonging the lives of recipients in their own homes, without impacting mortality. Furthermore, the integration of HCSs decreased the length of stays in any center by 7.7%, including hospitals, intermediate care centers, and nursing homes, and increased the use of day centers by 23.7%. Recipients of integrated HCSs had an increased probability of receiving social HCSs for household needs and decreased the use of the economic allowance for non-professional caregivers by 26.6%, with an increased utilization of primary care services. Despite the increased social expenditures associated with the initiation of social HCSs for dependent people, the integration of services reduced the increase by 19.1% and reduced the overall increase in social and health expenditures by 2.6% without additional healthcare expenditures.

The general goal of social HCSs for dependent people is to prolong the time individuals needing long-term care live independently in their homes [28]. Results from this study show that the integration of social and health HCSs resulted in improved outcomes by decreasing the risk of institutionalization and the length of stays at centers, including hospitals, intermediate care centers, and nursing homes. These results reflect a shift from institutionalization services to primary care services and day care centers, with a concomitant decrease in non-professional caregivers. The improved outcomes in individuals receiving integrated home-based care (i.e., prolonged stay at their homes after starting social HCSs) may reflect a better coverage of patients’ needs and represent a better quality and adequacy of services. Furthermore, these results indicate that integration of HCSs facilitates achieving the goals of the social HCS-dependency program and the model of home care integration of the Catalan government [17]. Remarkably, institutionalization decreased and the use of community services, with a lower cost than institutionalization facilities, increased. In this regard, intensifying community services, particularly primary care, may avoid institutionalization in areas with high integration in the near future.

Previous studies assessing the impact of health and social services integration following different schemes have provided evidence supporting improved administrative efficiency and patient experience [29,30]. A previous study in Scotland showed decreased rates of delayed discharges with no changes on mortality rates, similarly to this study [29]. Another study in England showed a modest impact on emergency admissions with no changes on days of hospitalization [30]. Systematic reviews focusing on the integration of home-based care programs for frail older people have shown decreased admissions to acute services and decreased risk of institutionalization [31]. However, systematic reviews of different care models for older persons reported increased use of services, with variable results regarding nursing home admissions [32]. Studies were highly heterogeneous regarding their design, data source, and outcome measures, determined by the main goals of the model assessed, precluding drawing strong conclusions. Moreover, differences in the study setting regarding the organization and nature of the healthcare systems further increased the heterogeneity [32]. Nevertheless, similarly to this study, previous studies agree that integrated care likely improved outcomes [31,33].

In the Catalan setting, small-scale interventions have been assessed. A post-stroke intensive home care program based on the integration of social and health care for improving home care of patients discharged from the hospital after a stroke shortened the time-to-service provision and increased the time to institutionalization [34]. Similarly to our study, this program showed an impact on institutionalization, but not on two-year death rates, suggesting a limited sensitivity of the mortality analyses.

An integrated provision of social and health HCSs is considered a cost-effective strategy, as it may prevent the duplication of services. Previous cost-effectiveness studies have typically focused on specific populations and conditions, but a recent systematic review and meta-analysis estimated a 5.6% decrease in health care-associated costs [33]. This meta-analysis showed decreased costs in studies lasting >12 months, which may explain the minimal differences observed in healthcare-associated expenditures one year after initiating social HCS-dependency (0.2%) in our study [33]. In contrast, social care-associated expenditures decreased after one year, despite the increased needs of the population included in this study (i.e., people with the official recognition of dependency). Even though long-term studies may be needed to capture the long-term economic impact of integrated care [33], our results support the cost-effectiveness of integrating care in the home setting. In this regard, a decrease in expenditures may require an intensification of community services, especially primary care.

The selection of cases and controls in this study was based on the results of a questionnaire assessing the level of integration of HCS developed in the context of the Integrated Care Program of Catalonia [21]. The evaluation framework established five core components of integrated care according to the conceptual framework of the Catalan model for integrated health and social home care [16,17]. This model was developed with the participation of over 70 stakeholder representatives, who performed a snowball literature review of over 300 documents to develop the model and the evaluation framework [20,21]. The concept and definition of integrated care is ambiguous and measuring successful care integration is still challenging, particularly in the home care setting, given the variety of health care and social services involved and the different public and private organizations providing them [20]. Moreover, a standardized tool to measure integrated care is still missing [35,36]. For this reason, the five core components of integrated care used to evaluate the level of integration had been selected through a consensus process considering available recommendations from international organizations [20].

The questionnaire assessing the level of integration was addressed to social care professionals working in SCS and PHCs of Catalonia, who were considered the reference professionals owing to their crucial role as coordinators of processes of integrated care [21]. The categorization of SCSs and PHCs into low/no integration and high integration was based on their perceptions. All SCSs professionals were invited to answer the questionnaire jointly with the PHCs in their area whenever possible, and all of them responded (100% response rate). Additionally, all PHCs were invited to answer the questionnaire, and 100 teams responded (25% of PHCs), reflecting an interest in the study and the integration of HCS, despite the tremendous workload of PHCs during the COVID-19 pandemic [21]. Nevertheless, the questionnaire’s responses were obtained across Catalonia, capturing the degree of integration in a widespread territory including urban and rural areas. Therefore, the methods used to assess integration allowed the stratification of a representative sample of SCSs and PHCs and a reliable selection of cases and controls.

Although the categorization of SCSs and PHCs into three levels of integration in this study may seem an oversimplification of the complexity of integrated care, our previous study analyzed numerous indicators and measurement elements within each of the core components [21]. However, the aim of this study was to assess the impact of integration, and three levels of integration were deemed sufficient to meet this goal. Future studies focused on specific components and elements may address their specific impact on health and social care outcomes.

The results from this study should be interpreted in the context of limitations associated with its retrospective observational design, which may have prevented establishing causality, and the use of routinely collected data from the CHSS and the SIDEP. In this regard, the information collected in these databases was limited, precluding the analysis of functional and quality-of-life outcomes, which are not systematically and homogeneously collected using patient-reported measures. Future analyses should include quality-of-life and functional assessment variables, which are not collected in administrative minimum data sets (MDS), only in the medical records. Furthermore, evaluating patient experience and satisfaction with the different HCSs received was unfeasible in this study, given that the surveys used to evaluate patients’ experience and satisfaction are not standardized across Catalonia. The development of a unique consensus measure of experience and satisfaction is a priority in the current integrated care work plan. Nevertheless, users’ preferences with the official recognition of dependency regarding integrated HCSs remained unknown in this study. Moreover, HCSs users are given the option of receiving an allowance to hire the preferred services. Future studies focusing on users’ experiences and satisfaction may provide useful information for improving HCSs and integration. This study aimed to assess the impact of integration, focusing on recipients of a public initiative for individuals with the official recognition of dependency and considered public services. However, individuals may complement the public services received with private services, resulting in an underestimation of the utilization of social HCSs. Furthermore, in Catalonia, persons are entitled to social care based on needs, but not so much on income, with no strict selection criteria, which differs among local governments. In contrast, in other European countries, entitlement to social care services is not strictly based on needs, but considers an individual economic assessment, which may result in a biased access to social care services, particularly in cases of high demand. In this regard, future studies assessing the integration of home-based services should also include private services and the associated expenditures incurred by the users. Likewise, although we acknowledge the importance of informal care, it was not considered in this study due to the lack of a database collecting this information. Additionally, this study focused on the population with the official recognition of dependency, likely needing long-term care, and excluded the population receiving other social HCSs, whose data are collected in multiple databases by local service providers. Moreover, previous studies recommend evaluations, especially economic ones, over long periods to capture all the relevant costs and benefits, and even lifetime evaluations [37]. In this regard, this study evaluated the impact of integrated care at one year, and the long-term effect of integrated care remained unassessed.

Despite its limitations, this study has strengths related to its design and the use of information from two databases. Considering that prospective controlled studies in this setting are unfeasible, the natural experiment approach with a differences-in-differences design using paired case-control data from population groups with and without integration allowed us to infer causality. In this regard, choosing the correct comparator for integrated care may be challenging, and “standard practice” (or usual care) is often selected as a comparator without controlling for partial or low-intensity integrated care [38]. In this study, we selected the comparator group (individuals with no integrated care) based on the same criteria used to define the population receiving integrated care, and we further performed a careful pairing of the two groups. This study, which integrated data from two administrative databases to analyze social and health data simultaneously, was unique considering the strict regulations preventing sharing data between different databases. Very few countries have integrated data from the health and social administrative databases. This study used population data, minimizing bias associated with sample selection and, consequently, allowing robust and precise estimation of the impact of integrated social and health HCSs.

Integrated care is considered a complex intervention, and its evaluation is challenging [38]. Nevertheless, this study integrating data from two administrative databases at the population level and using a case-control design captured the impact of integrating health and social HCSs in the Catalan setting. The results from this study support the cost-effectiveness of integrated care and provide useful information for the implementation of the Catalan model for integrated health and social home care, starting in 2020.

## Conclusions

This study evaluated the impact of integrated health and social HCSs in the population of Catalonia with the official recognition of dependency in 2019, before the implementation of the Catalan model for integrated health and social home care, starting in 2020. Integrated HCSs decreased the probability of institutionalization in nursing homes and decreased the length of stays at any center (acute care, mental health, intermediate care, and long-term care centers). Moreover, integration decreased the expenditures associated with social services, despite the increased expenditures associated with the initiation of social HCSs, resulting in a trend toward decreased total social and health expenditures. These results support the sustainability of integrated HCSs and their importance to prolong the lives of individuals needing long-term care in their homes. Moreover, they suggest that reinforced and intensified community services may avoid institutionalization. Given that the Catalan population is experiencing one of the most intensive ageing processes in the world, the Catalan Government should develop a policy and action plan, which may likely entail a reinforcement of health and social community services.

## Data Accessibility Statement

The datasets used and/or analyzed during the current study are available from the corresponding author upon reasonable request.

## Additional File

The additional file for this article can be found as follows:

10.5334/ijic.8909.s1 Supplementary information. Impact of Integrating Social and Health Home Care Services in Catalonia: A Retrospective Cohort-Based Two-Year Study. Assessment of the Integration Level/Degree.
