# Supplementary material for: Impact of Integrating Social and Health Home Care Services in Catalonia: A Retrospective Cohort-Based Two-Year Study
Source: Int J Integr Care. 2025 May 16;25(2):15. doi: 10.5334/ijic.8909 (PMC12083072; doi:10.5334/ijic.8909)
Supplement: Supplementary information. — Impact of Integrating Social and Health Home Care Services in Catalonia: A Retrospective Cohort-Based Two-Year Study. Assessment of the Integration Level/Degree. [file ijic-25-2-8909-s1.pdf]

Supplementary Information for the Submission by Vela *et al.* “Impact of Integrating Social and Health Home Care Services in Catalonia: A Retrospective Cohort-Based Two-Year Study.”

Supplementary Methods

Assessment of the Integration Level

The screening questionnaire used to assess the integration of social and health HCSs [1] included five items corresponding to the five core components of integrated care: 1) individual assessment of integrated social and health care; 2) single individual plan for integrated social and health care, built collaboratively and accessible by social and health care professionals; 3) shared protocols across health and social services; 4) coordination between social and health multidisciplinary teams; and 5) integrated portfolio services with joint social and health HCS projects [2]). The five items were answered on a 0 (absence of integration) to 5 (excellent integration) scale. The questionnaire was sent to all SCS coordinators (n=105) and all referents for social work at PHCs (n=376) in Catalonia, of which 105 (100%) and 94 (25.0%), respectively, provided answers [1]. SCSs with mean scores ≥2.5 (of a maximum of 5 points) for the five core components of integrated care regarding social services were considered to have a high level of integration. PHCs covering areas with excluded SCSs (i.e., mean scores <2.5) with mean scores ≥3.5 for the five core elements of integrated care regarding healthcare were considered to have a high level of integration. Conversely, SCSs with mean scores <2.5 were considered to have a low level of integration, and those with mean scores >2.5 and <5 were considered to have an intermediate level of integration (Figure S1).

Data Sources, Access, and Linkage Method

Sociodemographic and social services utilization data were obtained from the "Sistema per l'autonomia i l'atenció a la dependència" (SIDEP) (Autonomy and Dependency Care System) central registry. This database from the "Direcció General de

l’Autonomia Personal i la Discapacitat” (General Direction of Personal Autonomy and Disability) of the Social Rights Department collects personal data from individuals with the official recognition of dependency to manage dependency-associated procedures and develop an individual care plan. Data from individuals receiving other social services are collected in separate databases that are not centralized for the complete Catalan territory and consequently, recipients of other social home services outside the program for dependent people were excluded from this study.

The Catalan Health Surveillance System (CHSS) database collects detailed information regarding health care services utilization, including pharmacy, services and procedures, and diagnoses, including stratification according to the Adjusted Morbidity Group (GMA) from all the population of Catalonia [3].

Full access was granted to CHSS and its different data sources, and to the SIDEP registry. Person-level linkage across these data sources was performed using a personal identification code, which is an unequivocal code assigned to each person insured in the public healthcare system.

Variables

Income level was classified into four categories: high (annual income >€100 000), intermediate (€18 000–€100 000), low (<€18 000), and very low (receiving welfare support from the government), based on the income level categories used to establish pharmacy co-payments [4].

the type of municipality was classified as 1) urban (>20 000 inhabitants), 2) semi-urban (>10 000 inhabitants and >150 inhabitants/km^2), 3) semi-rural (>2000 inhabitants and between 100–150 inhabitants/km^2), and 4) rural (<200 inhabitants or <100 inhabitants/km^2) according to previously established definitions [5].

Clinical variables are recorded according to the usual clinical practice and coded following the International Classification of Diseases, ninth revision, Clinical Modification (ICD-9-CM) in the CHSS database [6].

The GMA assigns a numerical comorbidity index based on the type of disease (acute or chronic), number of systems affected, and complexity of each disease [7–9]. The GMA enables the classification into four levels of morbidity associated-risk: (1) baseline risk (healthy stage), with a GMA score up to the 50th percentile of the total population; (2) low risk, with a GMA score between the 50th–80th percentiles; (3) moderate risk, with a GMA score between the 80th–95th percentiles; and (4) high risk, with a GMA score above the 95th percentile [7–9].

Statistical Analysis

Categorical variables were described as frequencies and percentages, and quantitative variables, as the mean and standard deviation and the median and interquartile range (IQR: Q1, Q3). We used the McNemar and the Wilcoxon tests to compare paired categorical and continuous data, respectively, regarding the characteristics of cases and controls.

Supplementary Tables

Table S1. Definition of integrated social and health home care services based on the five core components of an integrated care model

Core components	Description
Individualized integrated social and health assessment.	An integrated and comprehensive assessment is carried out jointly using unique and shared tools between the two areas when integrated care is required.
Unique individual care plan.	A collaborative individual care plan is constructed, accessible to professional teams from both areas.
Shared protocols between health and social services.	Collaborative work dynamics with shared protocols between health and social services in different modalities (integrated care pathway, 24/7 care, management of risks in the home environment, etc.).
Coordination between multidisciplinary social and healthcare teams.	Spaces are created and time is dedicated to shared work between the two areas, reactively and with the capacity to respond to crisis situations, but also proactively, creating person-centered figures in both areas (reference and co-reference), and with the use of digital solutions that facilitate collaborative practice (integrated recording systems, shared messaging, etc.).
Integrated service portfolio with joint social and healthcare projects in the home environment.	Elements of the home care service portfolio are incorporated for common use and in service of both areas, facilitating direct access and prescription and activation of resources and services from each area that identifies the need.

Supplementary Figures

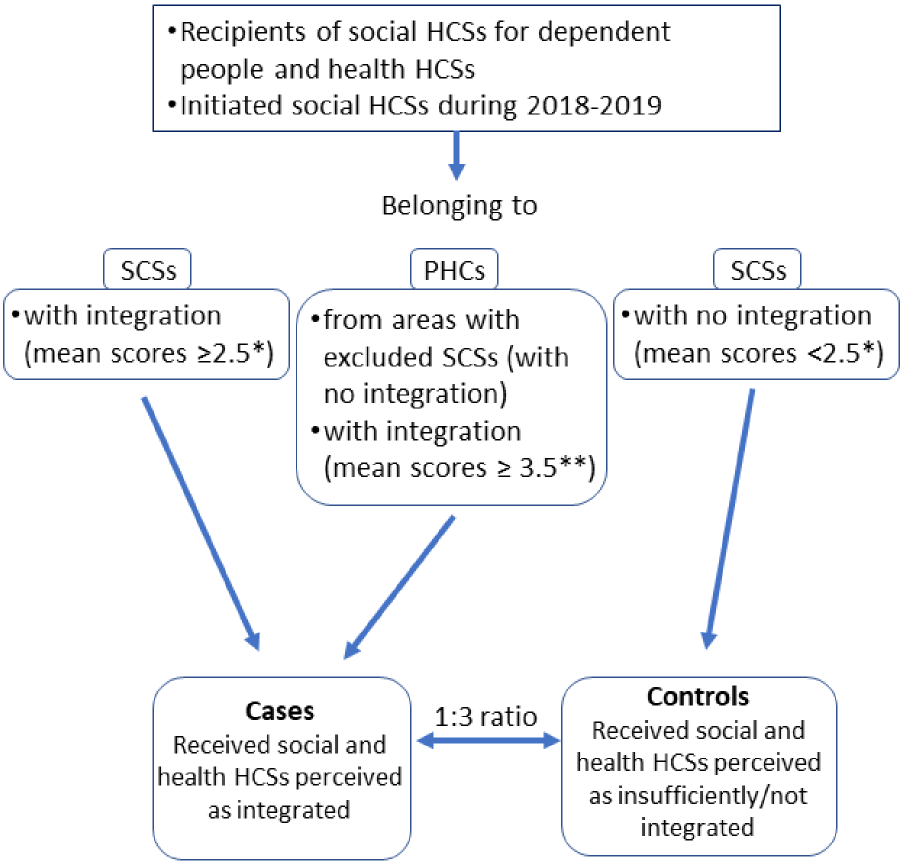

Figure S1. Diagram of the study design showing the selection of individuals and allocation to Case and Control Groups. HCSs, home care services; PHCs, primary health centers; SCSs, social care services.

Supplementary References

1. Hilarión P, Vila A, Contel JC, Santaeugènia SJ, Amblàs-Novellas J, Suñol R, et al. Integrated Health and Social Home Care Services in Catalonia: Professionals' Perception of its Implementation, Barriers, and Facilitators. Int J Integr Care [Internet]. 2024 [cited 2025 Feb 3];24(2):1–13. Available from: https://doi.org/10.5334/

2. General Directorate of Personal Autonomy and Disability D of SR. Avaluació de l'atenció integrada social i sanitària a l'entorn domiciliari. Context de referència i valoració de l'estat de la situació. (Evaluation of integrated social and health care. Context of reference and assessment of the state of the situation) [Internet]. 2021 [cited 2023 Sep 22]. Available from: https://dretssocials.gencat.cat/web/.content/01departament/05plansactuacio/avaluacions/1.2-Atencio-integrada-social-i-sanitaria-estudi-situacional.pdf

3. Amblàs-Novellas J, Santaeugènia SJ, Vela E, Clèries M, Contel JC. What lies beneath: a retrospective, population-based cohort study investigating clinical and resource-use characteristics of institutionalized older people in Catalonia. BMC Geriatr. 2020 Dec 2;20(1):187.

4. Bilal U, Cainzos-Achirica M, Cleries M, Santaeugènia S, Corbella X, Comin-Colet J, et al. Socioeconomic status, life expectancy and mortality in a universal healthcare setting: An individual-level analysis of >6 million Catalan residents. Prev Med (Baltim) [Internet]. 2019 Jun 1 [cited 2023 Jun 13];123:91–4. Available from: https://pubmed.ncbi.nlm.nih.gov/30853378/

5. Domínguez i Amorós M, Monllor i Rico N, Simó i Solsona M. Món rural i joves. Realitat juvenil i polítiques de joventut als municipis rurals de Catalunya.

Estudis: 31. Barcelona: Secretaria de Joventut. Generalitat de Catalunya; 2010. p. 1–113.

6. Centers for Disease Control and Prevention (CDC). ICD - ICD-9-CM - International Classification of Diseases, Ninth Revision, Clinical Modification. [Internet]. [cited 2025 Mar 4]. Available from: https://archive.cdc.gov/www_cdc_gov/nchs/icd/icd9cm.htm

7. Monterde D, Vela E, Clèries M, grupo colaborativo GMA. [Adjusted morbidity groups: A new multiple morbidity measurement of use in Primary Care]. Aten Primaria. 2016 Dec;48(10):674–82.

8. Monterde D, Vela E, Clèries M, Garcia-Eroles L, Roca J, Pérez-Sust P. Multimorbidity as a predictor of health service utilization in primary care: a registry-based study of the Catalan population. BMC Fam Pract. 2020;21(1):39.

9. Vela E, Clèries M, Monterde D, Carot-Sans G, Coca M, Valero-Bover D, et al. Performance of quantitative measures of multimorbidity: a population-based retrospective analysis. BMC Public Health. 2021 Dec;21(1).
